# p-STAT6, PU.1, and NF-κB are involved in allergen-induced late-phase airway inflammation in asthma patients

**DOI:** 10.1186/s12890-015-0119-7

**Published:** 2015-10-14

**Authors:** Deimante Hoppenot, Kestutis Malakauskas, Simona Lavinskiene, Raimundas Sakalauskas

**Affiliations:** Department of Pulmonology and Immunology, Medical Academy, Lithuanian University of Health Sciences, Kaunas, Lithuania; Laboratory of Pulmonology, Department of Pulmonology and Immunology, Medical Academ, Lithuanian University of Health Sciences, Kaunas, Lithuania

**Keywords:** Allergic asthma, Th9 cells, Interleukin-9, Eosinophil apoptosis, Transcription factors

## Abstract

**Background:**

Previous *in vitro* and animal studies demonstrated that transcription factors p-STAT6 and PU.1 are required to induce interleukin (IL)-9 secretion by T helper (Th) 9 cells. It is believed that n factor-kappaB (NF-κB) plays a role in eosinophil survival. The importance of these transcription factors in the pathogenesis of allergic asthma (AA) in humans is poorly understood. We evaluated p-STAT6 and PU.1 expression in peripheral blood Th9 cells and NF-κB expression in eosinophils during late-phase airway inflammation in AA patients.

**Methods:**

Nineteen adults with AA and 14 adult healthy individuals (HI) were examined. Peripheral blood collected 24 h before (baseline) and 24 h after bronchial allergen challenge. CD4^+^ cells and eosinophils were isolated by high-density gradient centrifugation and magnetic separation. The percentage of Th9 cells and apoptotic eosinophils was estimated by flow cytometry. p-STAT6 and PU.1 expression was expressed as mean fluorescence intensity (MFI) in Th9 cells. NF-κB levels were expressed as MFI in peripheral blood eosinophils. Serum IL-9 and IL-5 levels were determined by enzyme-linked immunosorbent assay.

**Results:**

At baseline, MFI of p-STAT6 and PU.1 in peripheral blood Th9 cells and MFI of NF-κB in eosinophils and, serum IL-5 and IL-9 levels were greater in AA patients (*P* < 0.05). Decreased eosinophil apoptosis was seen in the AA group compared with HI (*P* < 0.05). MFI of p-STAT6, PU.1, and NF-κB and serum levels of IL-5 and IL-9 were increased in the AA group 24 h after challenge compared with baseline (*P* < 0.05). In the AA group, a correlation between serum IL-9 and Th9 cells (*r* = 0.7, *P* = 0.001) and MFI of PU.1 (*r* = 0.6, *P* = 0.01) 24 h after bronchial allergen challenge was observed. A correlation between Th9 cells and MFI of p-STAT6 (*r* = 0.45, *P* = 0.03) as well as MFI of PU.1 (*r* = 0.5, *P* = 0.02) 24 h after challenge was only observed in AA patients. A correlation between the MFI of NF-κB and eosinophil apoptosis was observed in AA patients 24 h before (*r* = −0.46, *P* = 0.02) and after (*r* = −0.5, *P* = 0.02) challenge.

**Discussions:**

p-STAT6 and PU.1 may be associated with Th9 cells and IL-9 production, whereas NF-κB and IL-5 may be associated with reduced eosinophil apoptosis in allergen-induced late-phase airway inflammation.

**Trial registration:**

ClinicalTrials.gov NCT02214303

**Electronic supplementary material:**

The online version of this article (doi:10.1186/s12890-015-0119-7) contains supplementary material, which is available to authorized users.

## Background

The mechanisms of allergic asthma (AA) pathogenesis remain poorly understood despite modern investigational methods [1]. AA is a heterogeneous disease because the environmental stimuli, exposure to allergens, and involvement of different cells types and cytokines that participate in asthma pathogenesis are different between individuals. CD4^+^ T helper cells (Th) [2, 3] and eosinophils [4, 5] are involved in the pathogenesis of AA. The differentiation of CD4^+^ Th cells to different subsets depends on the type of cytokines present in the local environment and other stimuli. Interleukin-4 (IL-4) induces CD4+ T-cell proliferation and differentiation into Th2 cells that play an important role in asthma pathogenesis [6]. IL-12 induces the development of Th1 cells, which are important in cell-mediated immunity and phagocyte-dependent protective responses [7]. Transforming growth factor-β (TGF-β1), IL-6, IL-1β, and IL-23 are required for the differentiation of Th17 cells [8], which are associated with an increased number of airway neutrophils and a more severe asthma course. More recently, a newly identified T helper cell subset, Th9 cells, were identified and shown to develop under the influence of TGF-β and IL-4 stimuli [9, 10]. Th9 cells are thought to be an unstable subset that produce mainly IL-9 and are involved in allergic inflammation, some autoimmune diseases [11], and antitumor immune responses *in vivo* [12]. It was recently shown that Th9 cells differentiated from atopic children secrete greater amounts of IL-9 in bronchial lavage than those from non-atopic children [13]. Moreover, IL-9 is an important growth factor for repetitively stimulated T-cell lines and induces lung eosinophilia after allergen stimuli. Transcription factors often regulate the expression of different cytokines and are essential for the regulation of gene expression. Usually one or several specific transcription factors are key for subset-specific differentiation, and other transcription factors are shared between several subsets. Th9 cell development has been less studied than other CD4+ T helper subsets, but studies have shown that Th9 cells develop under stimulation of IL-4-activated signal transducer and activator of transcription protein-6 (STAT6). IL-4 and STAT6 are required to repress the expression of *Foxp3*, which induces a *Treg* phenotype and can repress IL-9 production [9, 10, 14]. STAT6 induces interferon regulatory factor 4 (IRF4) that is important for Th9 development [15]. Basic leucine zipper transcription factor is required for IL-9 production in both human and mouse Th9 cells and cooperates with IRF4 in binding to and activating the *Il9* locus [16]. IL-4 and STAT-6 activate the GATA3 gene, which is a main Th2 cell type regulator. E26 transformation-specific (ETS) family transcription factor PU.1 is a transcription factor that promotes switching between Th2 and Th9 phenotypes. PU.1 plays an important role in the development of Th9 memory cells and Th9 immunity [17].

Eosinophils develop from CD34+ pluripotent progenitor cells under certain stimuli and in many cases participate in the pathogenesis of AA. Progenitor CD34+ cells express high numbers of IL-5 receptors (IL-5R) and IL-5 generated by Th2 cells during asthma act on the bone marrow to enhance the production of eosinophils [18, 19]. Degranulated eosinophils release cytotoxic substances that damage airway epithelial cells [20]. IL-3, IL-5, and granulocyte-macrophage colony-stimulating factor prolong eosinophil survival *in vitro* by delaying apoptosis [21], while more recent studies have shown that IL-9 possesses eosinophil viability-enhancing effects [22]. Nuclear factor-kappaB (NF-κB) is believed to be an important transcription factor that mediates eosinophil survival because the inhibition of NF-κB in humans and animals induces eosinophil apoptosis [23].

This study investigated the role of the transcription factors PU.1, STAT-6, and NF-κB in allergen-induced inflammation in adult AA patients to try and mimic real-life conditions because the importance of these transcription factors in AA pathogenesis in humans has not been completely elucidated. The aim of this study was to evaluate STAT6 and PU.1 expression in peripheral blood Th9 cells and NF-κB expression in eosinophils during late-phase airway inflammation in AA patients after inhaled allergen bronchial challenge.

## Methods

The research protocol described below was approved by the Regional Biomedical Research Ethics Committee of the Lithuanian University of Health Sciences (BE-2-23). The study was registered in the U.S. National Institutes of Health trial registry ClinicalTrials.gov with identifier NCT02214303.

### Study population

We examined 33 nonsmoking adults (16 men and 17 women; median age, 26 years; interquartile range (IQR), 8) of which 19 patients had mild or moderate persistent AA and 14 were healthy individuals (HI). Asthma was diagnosed according to the revised 2012 GINA criteria [24]. All the study participants were recruited from the Department of Pulmonology and Immunology, Hospital of the Lithuanian University of Health Sciences, Kaunas. In all cases, informed consent was obtained using a written consent form and was signed by the study individuals. All participants were screened for inclusion and exclusion criteria. Inclusion criteria were patients with AA who had a clinical history of disease for ≥1 year, with current symptoms, and positive results of the skin prick test (≥3 mm) with *Dermatophagoides pteronyssinus* (*D. pteronyssinus*), birch pollen allergens, or five grass mixture allergens. All study subjects had refrained from using inhaled, nasal, or oral steroids for at least 1 month, long-acting *β*2 agonists for at least 48 h, and short-acting *β*2 agonists for at least 12 h before the lung function test. Use of antihistamines and antileukotrienes were stopped 7 days before the skin prick test and the lung function test. Baseline forced expiratory volume in 1 s (FEV_1_) was greater than 70 % of the predicted value in all patients. All healthy controls had normal spirometric values without airway hyperresponsiveness, no history of lung or other systemic immune diseases, and were nonatopic. None of the study participants had a history of smoking (Table 1).

**Table 1:** Demographic and clinical characteristics of study population

Characteristics	Allergic asthma patients *n* = 19	Healthy subjects *n* = 14
Age, median (range), years	25 (18–36)	27 (20–35)
Sex (male/female), n	10/9	7/6
Wheal diameter induced by allergen, median (range), mm	6.8 (5–11)	–
Sensitization to *D. pteronyssinus*/ birch/5 grass mixture allergen, n	12/4/3	–
PD_20_, geometric mean (range), mg	0.13 (0.03–0.42)	–
FEV_1_, mean ± SD (range), % of predicted	97 ± 10 (82–118)	102 ± 8 (95–122)
Maximum fall in FEV_1_ after bronchial allergen challenge during the first hour, median (range), %	27 (22–34)*	2 (3–8)

FEV_1_, forced expiratory volume in the first second

PD_20_, provocative dose of methacholine causing a 20 % fall in FEV_1_

*P < 0.05 versus healthy individuals

### Study design

**Fig. 1:**
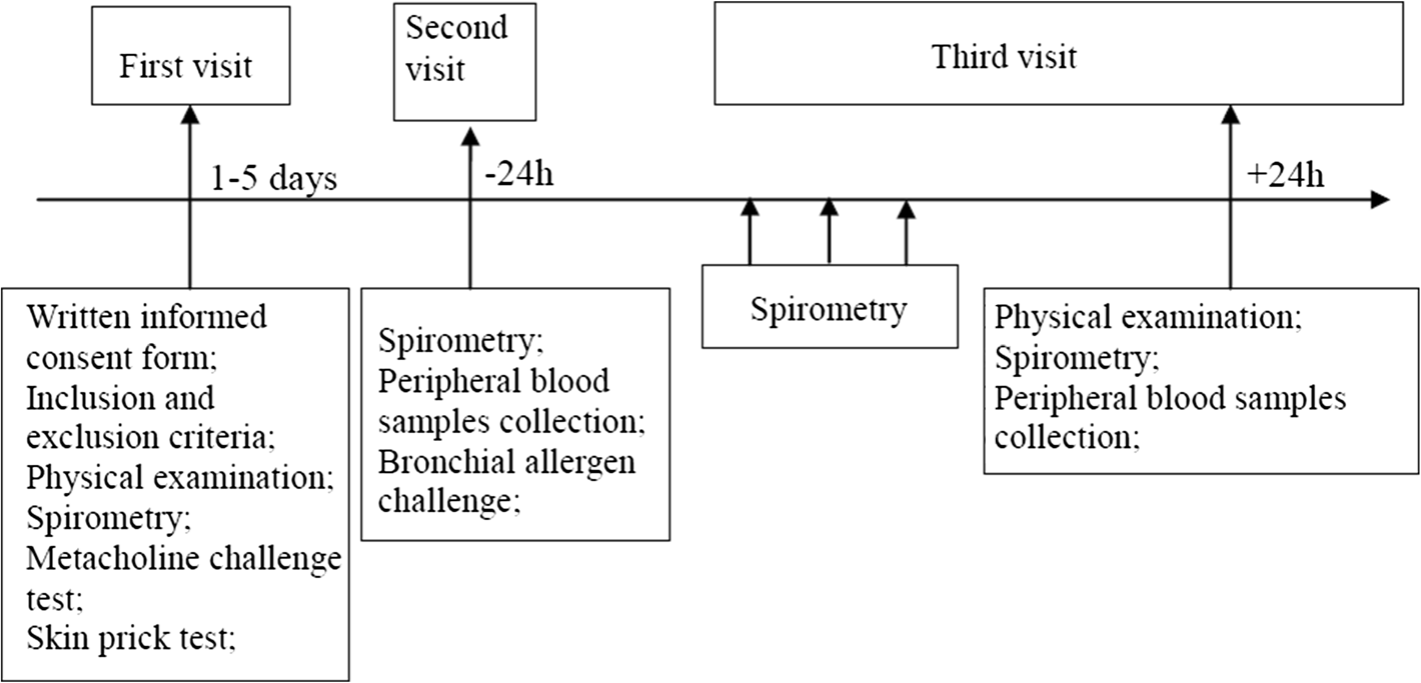
The flowchart of the study

Study individuals were seen at the screening visit where their eligibility for the study was checked based on inclusion and exclusion criteria. This visit included physical examination, spirometry, measurement of airway responsiveness to methacholine, and skin prick testing. At 24 h before bronchial allergen challenge, spirometry was performed and peripheral blood was collected from all eligible study subjects. An allergen (*D. pteronyssinus*, birch, or five grass mixture) for the bronchial challenge test in patients with AA was chosen according to the results of skin prick testing and clinical data in patients’ medical records. In the case of multiple positive results of the skin prick test, an allergen that most probably caused bronchoconstriction previously was chosen for specific bronchial challenge. All healthy volunteers were challenged with a *D. pteronyssinus* allergen. Bronchial allergen challenge was performed at 8:00 in the morning, and spirometry was reassessed every 10 min within the first hour and then every hour for the subsequent 6 h. Physical examination, spirometry, and peripheral blood collection were performed again 24 h after bronchial allergen challenge. A flow chart of the study is presented in Fig. 1.

### Measurement of airway responsiveness to methacholine

Methacholine was nebulized into a 10-L reservoir with a pressure nebulizer (Pari Provocation I; Pari, Stanberg, Germany). Aerolized methacholine was inhaled through a one-way valve at 5-min intervals starting with 15-μg methacholine dose and doubling it until a 20 % decrease in FEV_1_ from the baseline or the total cumulative dose of 3.87 mg was achieved. The bronchoconstricting effect of each dose of methacholine was expressed as a percentage of decrease in FEV_1_ from the baseline value. The provocative dose of methacholine causing a ≥ 20 % fall in FEV_1_ (PD_20_) was calculated from the log dose–response curve by linear interpolation of two adjacent data points [25, 26].

### Skin prick testing

Standardized *D. pteronyssinus*, *D. farinae*, cat and dog dander, 5 mixed grass pollen, birch pollen, mugwort, *Alternaria*, *Aspergillus*, and *Cladosporium* allergen extracts for skin prick testing were used (Stallergenes S.A., France) for all study individuals*.* Histamine hydrochloride (10 mg/mL) was used as a positive control, and physiological saline, as a negative control. After 15–20 min, the diameters of wheals were measured. The result was expressed as the mean of the longest diameter of the wheal and the longest diameter perpendicular to it. Reactions larger than 3 mm and at least half the size of that produced by histamine were considered as positive [27].

### Bronchial allergen challenge

Bronchial allergen challenge was performed with *D. pteronyssinus*, birch, or five grass mixture allergens (Stallergenes SA, Antony Cedex, France) at different concentrations (0.1, 1.0, 10.0, and 33.3 IR/mL) using a KoKo DigiDoser nebulizer (Sunrise Medical, Somerset, PA, USA) [28]. A freeze-dried lyophilized allergen was diluted with nonphenol saline. The results of the test were considered positive if there was a fall in FEV_1_ of 20 % or more from the baseline value. Spirometry was reassessed every 10 min within the first hour and then every hour for the subsequent 6 h (in case of a late asthmatic reaction occurrence). Spirometry was also performed 24 h after bronchial allergen challenge. For patients with pollinosis, bronchial allergen challenge was not performed during the birch pollen and grass pollen season.

### CD4+ T cells isolation from peripheral blood and intracellular pStat6 and PU.1 staining in Th9 cells

Peripheral blood lymphocytes were isolated from peripheral blood samples on Ficoll-Paque gradient and resuspended in RPMI 1640 (Sigma- Aldrich, USA) containing L-glutamine autologous plasma. CD4 T cells were separated from all mononuclear cells (PBMCs) using a magnetic separation kit (Miltenyi, USA). PBMCs were resuspended in cold MACS buffer (containing: PBS pH 7.2, 0.5 % bovine serum albumin [BSA] and 2 mM EDTA by diluting MACS BSA Stock Solution 1:20 in autoMACSR Rinsing Solution) (40 μL per 10^7^ total cells) and incubated with Biotin-Antibody Cocktail (CD8, CD14, CD15, CD16, CD19, CD36, CD56, CD123, TCRγ/δ, and CD235a (Glycophorin A) (10 μL per 10^7^ total cells) for 5 min. After incubation, 20 μL of Anti-Biotin MicroBeads (conjugated to monoclonal anti-biotin antibodies [isotype: mouse IgG1]) per 10^7^ total cells was added, mixed, and incubated for additional 10 min at 4 °C. A LS column (Miltenyi Biotec, USA) was prepared during this time by placing it in the magnetic field of MACS Separator and washing it with 2 mL of MACS buffer. A preseparation filter (30 μm Miltenyi Biotec, USA) was rinsed with MACS buffer and placed on the top of the column. The cells were then applied to the preseparation filter/LS column, and the magnetically labeled non-CD4 T cells depleted by retaining them on a column in the magnetic field of a Separator, while the unlabeled CD4 T cells passed through the column. The cell fraction was eluted with 5 mL of MACS buffer.

Cell fractions were centrifuged (400 *g*, 10 min, 4 °C), and the pellet resuspended in 1 mL of RPMI 1640. Two million cells per mL were activated for 5 h at 37 °C with phorbol-12-myristate-13-acetate (Sigma-Aldrich) and ionomycin (Invitrogen, USA) in the presence of a protein transport inhibitor (BD GolgiStopTM, BD Biosciences, USA) to avoid cytokine secretion. After the activation, the cells were fixed with BD CytofixTM fixation buffer (BD Biosciences). Permeabilization of the fixed cells was performed by adding BD Perm/WashTM buffer (BD Biosciences) and stained by adding human IFN-GMA Alexa 488, TGF-β Alexa 488, IL-17A Alexa 488, IL-4 Alexa 488, IL-9 PERCP-CY5.5, pStat6 PE, PU.1 primary antibody, and secondary IgG2a + b PE. Flow cytometry was performed on a FACSCalibur flow cytometer (BD Biosciences). Results are reported as the mean fluorescence intensity (MFI) of intracellular p-STAT6 and PU.1 in Th9 cells (CD4^*+*^ IL-9^+^ IFNγ^−^ TGFβ_1_^−^ IL-4^−^ IL-17^−^ cells).

### Eosinophil isolation from peripheral blood and intracellular NF-ĸB staining

Peripheral blood samples (20 mL) were diluted by adding the same volume of PBS. The suspension was carefully layered over Ficoll-Paque (ρ = 1.077 g/mL) in conical tubes and centrifuged at 1000 *g* for 30 min at 20 °C in a swinging bucket rotor without brake. A layer with mononuclear cells (PBMCs) was removed and used for Th cell isolation. Granulocytes were separated by hypotonic lysis of erythrocytes. Later, the granulocyte pellet was resuspended in cold MACS buffer (containing: PBS pH 7.2, 0.5 % BSA, and 2 mM EDTA by diluting MACS BSA Stock Solution 1:20 in autoMACSR Rinsing Solution) (40 μL per 10^7^ total cells) and incubated with Biotin-Antibody Cocktail (biotin-conjugated monoclonal antibodies against CD2, CD14, CD16, CD19, CD56, CD123, and CD235a (Glycophorin A) (10 μL per 10^7^ total cells) for 10 min. After incubation, 20 μL of Anti-Biotin MicroBeads (conjugated to monoclonal anti-biotin antibodies [isotype: mouse IgG1]) per 10^7^ total cells was added, mixed, and incubated for additional 15 min at 4 °C. A LS column (Miltenyi Biotec, USA) was prepared during this time by placing it in the magnetic field of MACS Separator and washing it with 2 mL of MACS buffer. A preseparation filter (30 μm Miltenyi Biotec, USA) was rinsed with MACS buffer and placed on the top of the column. The cells were then applied to the preseparation filter/LS column, and the magnetically labeled non-eosinophils depleted by retaining them on a column in the magnetic field of a Separator, while the nonlabeled eosinophils passed through the column. The cell fraction was eluted with 5 mL of MACS buffer.

Cell fractions were centrifuged (400 *g*, 10 min, 4 °C), and the pellet resuspended in 1 mL of RPMI 1640. Two million cells per mL were activated for 5 h at 37 °C with phorbol-12-myristate-13-acetate (Sigma-Aldrich) and ionomycin (Invitrogen, USA) in the presence of a protein transport inhibitor (BD GolgiStopTM, BD Biosciences, USA) to avoid cytokine secretion. After the activation, the cells were fixed with BD CytofixTM fixation buffer (BD Biosciences). Permeabilization of the fixed cells was performed by adding BD Perm/WashTM buffer (BD Biosciences) and stained by adding 20 μL of Alexa Fluor® 647 Mouse anti-NF-κB p65 (pS529) from BD, cat.no 558422 which recognizes the phosphorylated serine 529 (pS529) in the transactivation domain of the human NF-κB p65 subunit. NF-κB in general is an ubiquitously expressed transcription factor. Optimal activation of NF-κB requires phosphorylation (e.g. serine phosphorylation) in the transactivation domain of p65.

Flow cytometry was performed on a FACSCalibur flow cytometer (BD Biosciences). Results are reported as the MFI of intracellular NF-ĸB expression in peripheral blood eosinophils.

The peripheral blood cell analysis was performed on an automated hematology analyzer (Sysmex XE-5000, Japan) at the Department of Laboratory Medicine, Hospital of Lithuanian University of Health Sciences Kaunas Clinics.

### Detection of cytokines in serum

Serum cytokine levels were measured by the enzyme-linked immunosorbent assay (ELISA) according to the manufacturer’s instructions: the minimal detectable doses of IL-9 and IL-5 were 0.1 ng/mL (Abcam, USA) and 5 pg/mL (Abcam, USA), respectively.

### Apoptosis assay

Isolated eosinophils were resuspended in the annexin-binding buffer (pH 7.4) containing 50 mM HEPES, 700 mM NaCl, and 12.5 mM CaCl_2_ (Invitrogen, USA) and incubated with fluorescein isothiocyanate-labeled (FITC)-annexin V (Invitrogen, USA) and propidium iodide (PI) for 15 min at room temperature in the dark. After the incubation, apoptosis was analyzed by flow cytometry using the CellQuest software (BD Biosciences, USA). Apoptotic cells were quantified as the percentage of the total population that was positive for FITC, but negative for PI. Necrotic cells were positive for PI.

### Statistical analysis

Statistical analysis was performed by using the Statistical Package for the Social Sciences, version 20.0 for Windows (IBM SPSS Statistics 20.0, USA). The normality assumption of data was verified with the Shapiro–Wilks test. All the data were distributed not normally and were presented as median and ranges or interquartile range. Because of a skewed distribution of the variables, nonparametric tests were used. We analyzed two dependent groups: one group comprised the same allergic asthma patients 24 h before and 24 h after bronchial allergen challenge; the second dependent group consisted of the same healthy control individuals 24 h before and 24 h after bronchial allergen challenge. The changes in data before and after bronchial challenge in dependent groups were evaluated for statistical significance by the Wilcoxon test for paired analyses. Differences between two independent groups were evaluated using the Mann–Whitney U test. The Spearman rank test was used to assess relationships between variables. Statistical significance was assumed when *P* <0.05.

## Results

### Characteristics of study population

No significant age and sex differences were documented when both groups were compared. Only AA patients showed positive skin prick test results. In the AA group, 12 patients were sensitized to *D. pteronyssinus,* four patients to birch pollen allergens, and three patients to five grass mixture allergens. Five patients had positive skin prick test results for several inhalant allergens: birch pollen, five grass mixture allergens, mugwort, and *Alternaria*. Three patients had positive skin prick test results for *D. pteronyssinus* and five grass mixture allergens. Demographic and clinical data of the study population are presented in Table 1. Baseline FEV_1_ (% of predicted) did not differ when comparing both groups. A significant reduction in FEV_1_ after bronchial allergen challenge during the first hour was only observed in AA patients.

At the baseline, AA patients had a significantly higher peripheral blood eosinophil count compared with the HI group (*P* < 0.01). The absolute peripheral blood eosinophil number significantly increased in the AA group 24 h after bronchial challenge compared with the baseline values (*P* < 0.05). There were no significant differences in peripheral blood leukocyte, lymphocyte, and neutrophil counts before and after bronchial challenge when comparing the AA and HI groups (Additional file 1: Table S1).

### Peripheral blood Th9 cells and serum IL-9

**Fig. 2:**
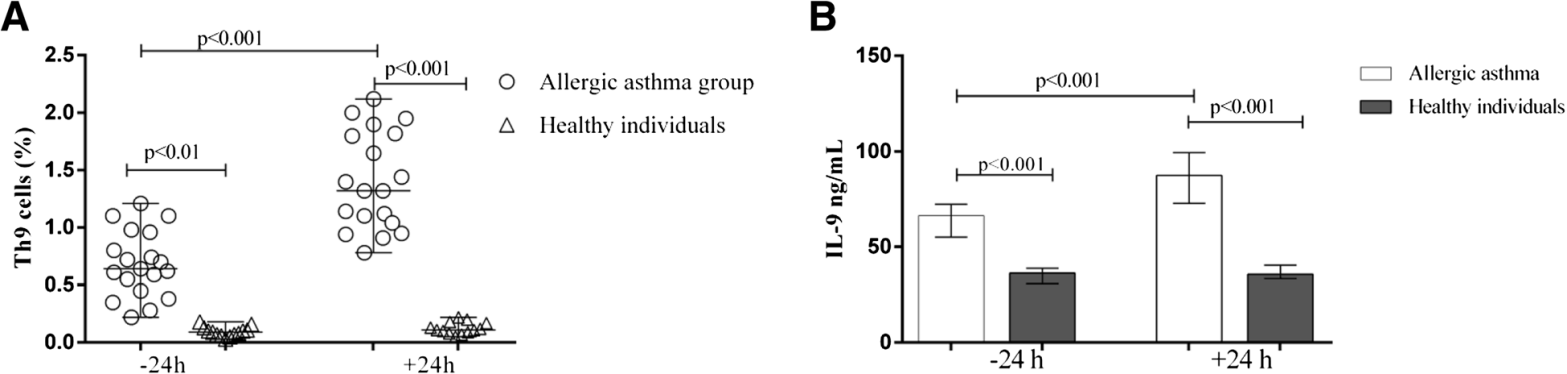
Percentage of peripheral blood Th9 cells (**a**) and serum IL-9 concentration (**b**) in patients with allergic asthma and healthy individuals 24 h before and 24 h after bronchial allergen challenge. Data are shown as median with minimum-maximum values (**a**) and as median with IQR (**b**)

At 24 h after bronchial allergen challenge, the percentage of Th9 cells (Additional file 2: Figure S1) was higher in the AA group compared with the control group and baseline values (1.32 % [0.78–2.12 %] vs. 0.11 % [0.05–0.22 %] and 0.64 % [0.22–1.21 %], respectively, *P* < 0.05) (Fig. 2a). At the baseline, the percentage of Th9 cells was greater in the AA group compared with the HI group (*P* < 0.05). There was no significant difference in the percentage of Th9 cells in the HI group before or after bronchial allergen challenge.

At the baseline, the serum IL-9 level was significantly higher in the AA than the HI group (66.5 ng/mL [31.3–82.4] vs. 36.3 ng/mL [28.2–42.9], respectively, *P* < 0.05). At 24 h after bronchial allergen challenge, the serum IL-9 level was significantly increased in the AA group compared with the baseline value and the HI group (87.4 ng/mL [51.41–114.2] vs. 66.5 ng/mL [31.25–82.4] and 38.9 ng/mL [32.6–46.3], respectively, *P* < 0.01) (Fig. 2b). In the AA group, the percentage of Th9 cells and serum IL-9 levels correlated with those at 24 h before (*r* = 0.52, *P* = 0.02) and 24 h after (*r* = 0.69, *P* = 0.001) bronchial allergen challenge.

### STAT6 and PU.1 transcription factors in Th9 cells

**Fig. 3:**
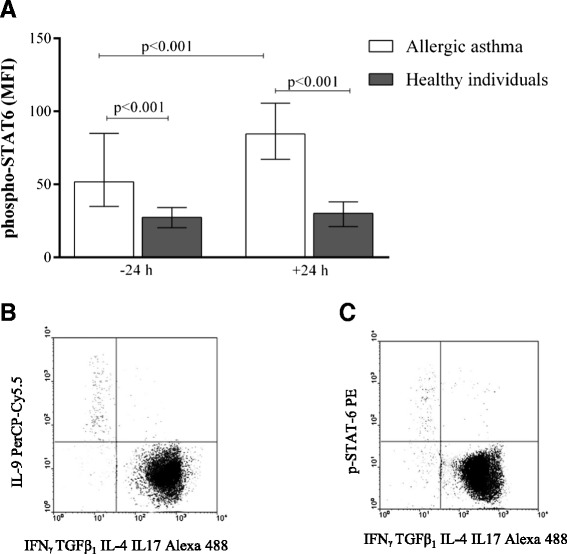
Diagram (**a**) represents the mean fluorescence intensity of p-STAT6 in Th9 cells in peripheral blood CD4^+^ cells in allergic asthma and healthy individuals 24 h before and 24 h after bronchial allergen challenge. Data are shown as median (minimum-maximum values). Dot plot (**b**) represents Th9 expression and dot plot (**c**) represents p-STAT6 expression in Th9 cells in one allergic asthma patient after bronchial allergen challenge

**Fig. 4:**
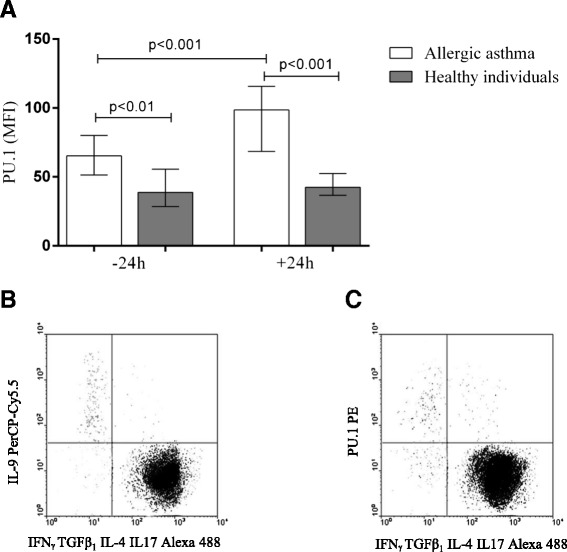
Diagram (**a**) represents the mean fluorescence intensity of transcription factor PU.1 in Th9 cells among peripheral blood CD4^+^ cells in allergic asthma and healthy individuals before and after bronchial allergen challenge. Data are shown as median (minimum-maximum values). Dot plot (**b**) represents Th9 expression and dot plot (**c**) represents PU.1 expression in Th9 cells in one allergic asthma patient after bronchial allergen challenge

At 24 h after bronchial allergen challenge, there was a significantly higher mean MFI of p-STAT6 in Th9 cells in the AA group compared with the baseline value and the HI group (84.6 MFI [67.2–105.5] vs. 51.7 MFI [34.9–84.9] and 30.2 MFI [21.1–38.12], respectively; *P* < 0.01). The mean MFI of p-STAT6 was also higher in the AA group compared with the HI group at the baseline (51.7 MFI [34.9–84.9] vs. 27.4 MFI [20.3–34.2], *P* < 0.01) (Fig. 3a–c). At 24 h after bronchial allergen inhalation, the MFI of PU.1 in Th9 cells was higher compared with the baseline data and the HI group (98.6 MFI [68.45–115.68] vs. 65.2 MFI [51.3–80.1] and 42.4 MFI [36.51–52.4], respectively; *P* < 0.01). The mean MFI of PU.1 remained unchanged after bronchial allergen challenge in the HI group (38.7 MFI [28.4–55.6] vs. 42.4 MFI [36.5–52.4], respectively; *P* > 0.05) (Fig. 4a–c). The percentage of Th9 cells correlated with p-STAT6 (*r* = 0.45, *P* = 0.03), and the serum IL-9 level correlated with PU.1 (*r* = 0.6, *P* = 0.01) and the percentage of Th9 cells (*r* = 0.7, *P* = 0.001) after bronchial allergen challenge in the AA group only. Moreover, there was a significant correlation between PU.1 and Th9 cells in the AA group 24 h after allergen challenge (*r* = 0.5, *P* = 0.02). There was no significant correlation between the percentage of Th9 cells and serum IL-9 level or p-STAT6 and PU.1 in the HI group.

### Peripheral blood eosinophil apoptosis, serum IL-5, and NF-κB expression

Serum IL-5 levels were significantly greater in the AA group than the HI group 24 h before bronchial allergen challenge (42.3 pg/mL [28.7–55.6] vs. 16.2 pg/mL [14.2–18.5], *P* < 0.01) (Additional file 3: Figure S2). At 24 h after bronchial allergen challenge, serum IL-5 levels were significantly increased in the AA group compared with the baseline value and the HI group (78.9 pg/mL [54.9–90.2] vs. 42.3 pg/mL [28.7–55.6] and 16.2 pg/mL [14.6–19.3], respectively; *P* < 0.01). There was no significant difference in the serum IL-5 level before and after bronchial allergen challenge in the HI group.

**Fig. 5:**
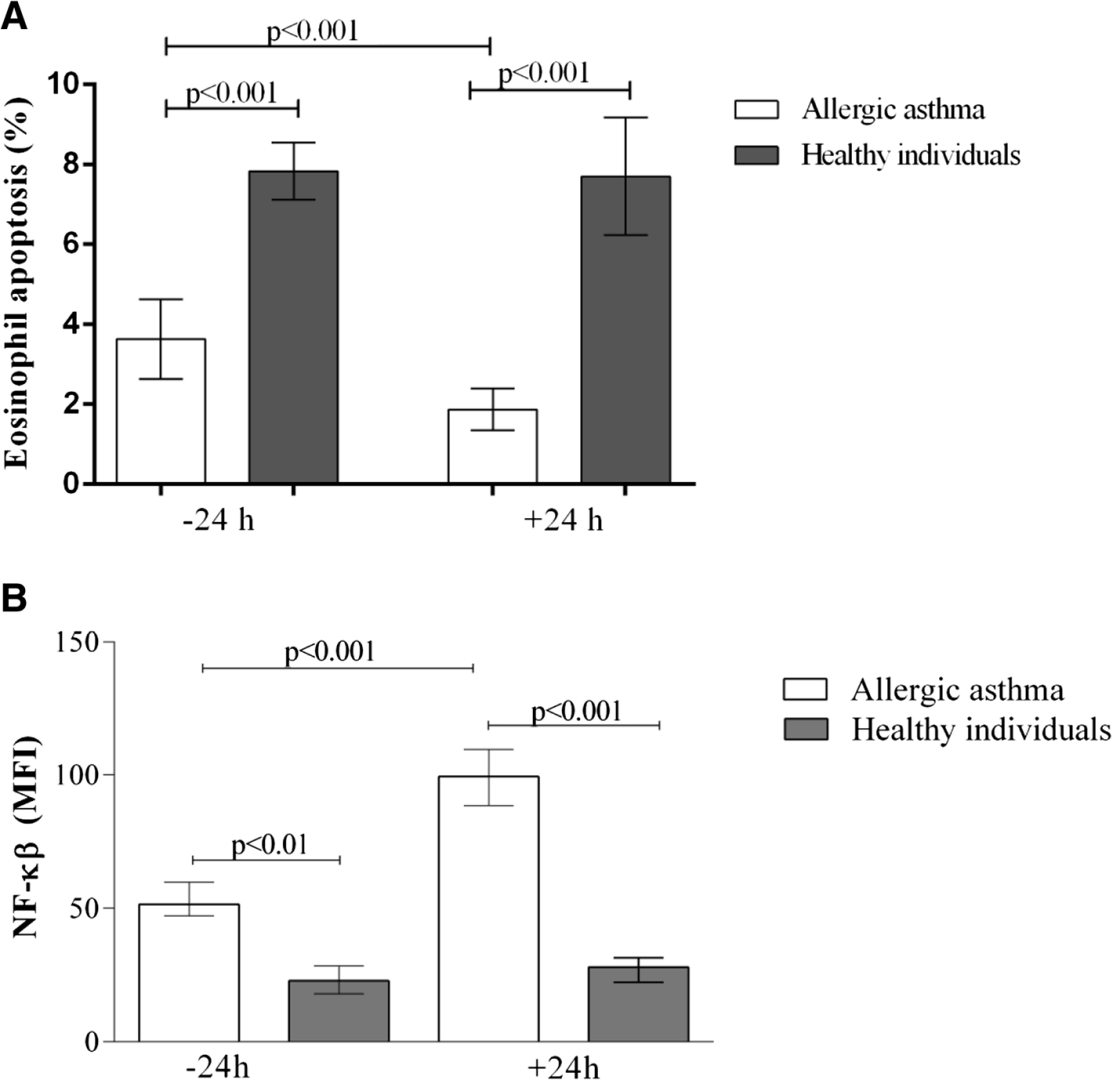
Peripheral blood eosinophil apoptosis (**a**) and mean fluorescence intensity of NF-ĸB in peripheral blood eosinophils (**b**) in patients with allergic asthma and healthy individuals before and after bronchial allergen challenge. Data are shown as median with minimum-maximum values (**a**) and median with IQR (**b**)

At the baseline, a significantly lower percentage of peripheral blood apoptotic eosinophils was observed in the AA group compared with the HI group (3.45 % [2.1–5.4 %] vs. 7.9 % [5.0–10.2 %] *P* < 0.001). At 24 h after bronchial allergen provocation, the percentage of apoptotic eosinophils decreased in the AA group compared with the baseline value and the HI group (2.2 % [0.9–3.12 %] vs. 3.5 % [2.1–5.4 %] and 8.0 % [6.6–9.1 %], respectively; *P* < 0.001). Eosinophil survival in the HI group remained the same even 24 h after bronchial allergen challenge (Fig. 5a). Correlation between the percentage of apoptotic peripheral blood eosinophils and serum IL-5 levels was only observed in AA patients 24 h after bronchial allergen challenge (*R* = −0.78, *P* = 0.03), but there was no significant correlation in this group 24 h before allergen bronchial challenge. In addition, we did not observe a correlation between the percentage of apoptotic peripheral blood eosinophils and serum IL-5 levels in the HI group at any time point.

Our data showed a greater MFI of NF-κB in eosinophils in the AA group 24 h after bronchial allergen challenge compared with the baseline value and the HI group (99.3 MFI [79.5–153.5] vs. 51.4 MFI [39.2–70.26] and 27.8 MFI [17.2–34.15], respectively; *P* < 0.001) (Fig. 5b). There was no change in NF-κB in the HI group. Furthermore, we observed significant negative correlations between the MFI of NF-κB and eosinophil apoptosis 24 h before (*r* = −0.52, *P* = 0.02) and after (*r* = −0.46, *P* = 0.02) allergen provocation.

## Discussion

Our study has shown that p-STAT6 and PU.1 may be important for Th9 cells and IL-9 production, whereas NF-κB and IL-5 were associated with reduced eosinophil apoptosis in allergen-induced late-phase airway inflammation in AA patients.

A decade ago it was assumed that IL-4 and TGF-β could induce IL-9 production from CD4+ T cells [29], and this theory was proved by two independent groups [9, 10]. Only a few CD4+ T-cell subsets are stable; often, CD4+ T-cell subsets acquire new characteristic features typical of different phenotypes dependent upon the type of stimulating cytokine, and one polarized phenotype can switch to another polarized phenotype [30]. Th9 cells are a CD4+ cell subset with great plasticity and flexibility. Th9 cells require both IL-4 and TGF-β stimulation because IL-4 alone stimulates the development of CD4^+^ T cells into Th2 cells, and TGF-β alone induces *Treg* cells. Th9 cells have lower *Foxp3* expression than *Treg* cells, lack Th2 cytokine production, and produce high levels of IL-9. The plasticity of CD4+ T cells might be explained by the presence of specific transcription factors [31]: Th2 and Th9 cell phenotypes share common factors such as STAT6, PU.1, GATA3, c-maf, and IRF4. Environmental factors activate the expression of only one or several transcription factors, which induce another CD4+ T-cell phenotype. Although the number of Th9 cells is low and Th9 cells are closely related to Th2 cells, these cells can be considered a unique and separate CD4^+^ T-cell subset. There is a lack of studies and consensus regarding Th9 cells; therefore, further studies on this new cell subset, especially in humans, are needed. Many studies have used mouse models, which are useful for the investigation of responses to inhaled allergens. Murine models allow the application of a broad range of immunological tools as well as gene deletion *in vivo.* Studies of Th9 cells showed that both human and murine Th9 cells do not express cytokines characteristic of other CD4+ T-cell subsets except for IL-9; however, some authors believe Th9 cells also produce IL-10. However, in contrast to murine Th9 cells, IL-10 is not expressed by human Th9 cells [10, 32, 33]. Other studies have suggested the importance of Th9 cells in airway allergic inflammation [14, 34–36], although some issues remain to be resolved. To study Th9 cells and their transcription factors as well as the role of eosinophils in AA, we decided to challenge study individuals with an inhaled specific allergen as this provocation model imitates natural allergen exposure in humans.

There is some evidence that PU.1, a TS-family transcription factor, is required for the generation of Th9 cells. In the study by Chang et al., naive T cells from wild-type and PU.1 knockout mice were differentiated under conditions favorable for the development of Th9 cells [36]. It was found that IL-9 protein secretion, IL-9 mRNA expression, and IL-9 intracellular staining were decreased in Th9 cultures that lacked PU.1 expression. This abovementioned study showed that PU.1 bound to sites in the *Il9* promoter specifically in Th9 cells and promoted the switch between Th2 and Th9 phenotypes. PU.1 protein is encoded by the *SPI1* gene in humans, and in the same study, the inhibition of *SPI1* expression in human Th9 cell cultures resulted in a significantly lower production of IL-9 [36]. To identify associations between human Th9 cells and PU.1, we applied the bronchial challenge test to AA patients using an inhaled specific allergen as this provocation model imitates natural allergen exposure quite well. We investigated PU.1 expression using flow cytometry analysis of Th9 cells from human peripheral blood and found a higher expression of this transcription factor in AA patients compared with the control group, and the expression was more prominent 24 h after allergen exposure. Moreover, the MFI of PU.1 only correlated well with the percentage of Th9 cells and serum IL-9 levels in the AA group 24 h after bronchial allergen challenge. These data support the findings from other studies that PU.1 is important in Th9 cell differentiation.

STAT6 belongs to the family of transcription factors that activate gene transcription in response to a particular cytokine. It is a cytoplasmic protein that is activated by tyrosine phosphorylation by cytokine receptor-associated JAK (Janus) kinases after cytokine exposure. IL-4 activates STAT6 and the expression of a very specific gene program that regulates viability, proliferation, and differentiation of the responding cell. STAT6 only recognizes phosphorylated tyrosines on the cytoplasmic tail of the IL-4 receptor α chain and does not interact with phospho-tyrosines on the IFN-γ receptor [37]. JAK kinases, Jak1 and Jak3, bind to the α and γ IL-4R chains, respectively, and are responsible for the subsequent phosphorylation of the IL-4 receptor itself as well as STAT6. The phosphorylated STAT6 monomers then dimerize, translocate to the nucleus, and bind DNA to activate transcription [38]. STAT6 is required for the modification of TGF-β-induced *Treg* development because Th9 cells develop when TGF-β and IL-4 act together. Mice expressing constitutively active STAT6 are predisposed to allergic disease [39]. We found greater pSTAT6 expression in IL-9-producing cells from AA patients compared with the HI group. A correlation between serum IL-9 levels and pSTAT6 as well as between the percentage of Th9 cells and pSTAT6 indicated the importance of this transcription factor in the development and function of Th9 cells.

IL-9 is a signature cytokine of Th9 cells. Th2 cells, mast cells, natural killer cells, Th17 cells, and Treg cells under some conditions produce less clinically important amounts of IL-9. It was shown that IL-9 increased the expression of IL-5R on eosinophils and inhibited eosinophil apoptosis, enhancing eosinophil development and promoting eosinophil maturation in synergy with IL-5 [22, 39]. Expression of IL-9 in transgenic mice under the control of a lung-specific promoter resulted in severe airway inflammation with eosinophils and lymphocytes as well as mast cell hyperplasia [40]. We investigated serum IL-9 levels in patients with AA and HI before and after allergen challenge and found higher serum IL-9 levels in patients with AA, which increased further after allergen provocation. The IL-9 level correlated with the percentage of peripheral blood Th9 cells 24 h after allergen challenge in AA patients. These findings indicate the important role of IL-9 and Th9 cells in allergen-induced late-phase airway inflammation.

TGF-β-, IL-4-, and T-cell receptor-induced transcription factors (including NF-κB non-canonical pathway) co-stimulate acute *Il9* expression. The *Il9* promoter region has multiple NF-κB binding sites [31, 41]. Moreover, TGF-β activates NF-κβ, which reduces eosinophil apoptosis and in this way prolongs eosinophil survival [42, 43]. In a recent study by Kankaanranta et al., cultures of peripheral blood eosinophils from healthy, atopic, or asthmatic donors (patients were allowed to continue usual treatment with inhaled steroids or antihistaminic drugs) were investigated, and the data demonstrated a major role for NF-κB in the TNF-β-induced inhibition of eosinophil apoptosis [44]. Inhibition of NF-κB leads to decreased airway eosinophilia in mice [45]. As delayed eosinophil apoptosis in asthma is a major cause of prolonged eosinophil inflammation [46, 47], we investigated peripheral blood eosinophil apoptosis 24 h before and after bronchial allergen challenge. We found a significantly higher percentage of apoptotic eosinophils and higher serum IL-5 levels in the AA group compared with the HI group at the baseline. However, the percentage of apoptotic eosinophils correlated with NF-κB expression in the AA group 24 h after bronchial allergen challenge but did not correlate with the IL-5 levels. Serum IL-5 levels were also increased in AA patients after challenge. The HI group did not show any significant changes in serum IL-5 levels or peripheral blood eosinophil apoptosis. Our data support evidence from previous studies that NF-κB and IL-5 are important in prolonging eosinophil survival.

## Conclusions

In summary, our data show associations between p-STAT6, PU.1, and Th9 cells in adult AA patients. In addition, associations between eosinophil NF-κB, serum IL-5, and peripheral blood eosinophil apoptosis in late-phase airway inflammation in AA patients were observed.

## Additional files

Additional file 1: Table S1. Changes in peripheral blood cells before and after bronchial allergen challenge in allergic asthma patients and healthy individuals. (DOC 34 kb) Additional file 2: Figure S1. Dot plot representing lymphocytes by size and granularity (A); T lymphocytes gated by expressing CD4^+^ (B); Th9 expression (C). (DOC 113 kb) Additional file 3: Figure S2. The serum IL-5 concentration in patients with allergic asthma and healthy individuals before and after bronchial allergen challenge. (DOC 76 kb)

## Abbreviations

AA: Allergic asthma
STAT-6: Signal transducer and activator of transcription protein-6
NF-ĸB: Nuclear factor kappa-light-chain-enhancer of activated B cells
IL: Interleukin
Th: T helper lymphocyte
IFN-*γ*: Interferon gamma
TGF-*β*: Transforming growth factor beta
FEV_1_: Forced expiratory volume in one second
IL-5R*α*: IL-5 receptor *α*-chain
FVC: Forced vital capacity
HI: Healthy individuals
PBMCs: Peripheral blood mononuclear cells
GM-CSF: Granulocyte-macrophage colony-stimulating factor
EDTA: Ethylenediaminetetraacetic acid
