# Supplementary material for: p-STAT6, PU.1, and NF-κB are involved in allergen-induced late-phase airway inflammation in asthma patients
Source: BMC Pulm Med. 2015 Oct 14;15:122. doi: 10.1186/s12890-015-0119-7 (PMC4606997; doi:10.1186/s12890-015-0119-7)
Supplement: Additional file 1: Table S1. — Changes in peripheral blood cells before and after bronchial allergen challenge in allergic asthma patients and healthy individuals. (DOC 34 kb) [file 12890_2015_119_MOESM1_ESM.doc]

| Characteristics | 24 h before bronchial allergen challenge | 24 h after bronchial allergen challenge |
| --- | --- | --- |
| Allergic asthma patients | | |
| Leukocytes, ×10^9^/L | 5.9 (4.7–11.2) | 6.3 (4.8–12.1) |
| Eosinophils, ×10^9^/L | 0.2 (0.11–0.88) ^#^ | 0.52 (0.09–1.19)*^#^ |
| Eosinophils, % | 4.7 (0.5–10.4) ^#^ | 7.2 (1.6–12.1)* ^#^ |
| Neutrophils, ×10^9^/L | 3.2 (2.6–7.6) | 3.0 (2.1–9.5) |
| Neutrophils, % | 50.4 (44.2–69.4) | 49.3 (36.4–74.8) |
| Lymphocytes, ×10^9^/L | 2.11 (1.52–3.44) | 2.2 (1.3–2.8) |
| Lymphocytes, % | 31.3 (21.5–42.3) | 32.5 (21.9–43.3) |
| Healthy individuals | | |
| Leukocytes, ×10^9^/L | 6.0 (4.15–9.01) | 6.1 (4.22–9.2) |
| Eosinophils, ×10^9^/L | 0.14 (0.03–0.88) | 0.13 (0.08–1.2) |
| Eosinophils, % | 2.8 (0.7–8.4) | 2.7 (1.1–9.6) |
| Neutrophils, ×10^9^/L | 2.85 (2.29–5.96) | 3.02 (2.34–5.59) |
| Neutrophils, % | 54.1 (35.6–70.2) | 55.2 (38.6–60.8) |
| Lymphocytes, ×10^9^/L | 2.32 (1.11–3.24) | 2.04 (1.19–3.45) |
| Lymphocytes, % | 37.9 (16–59.6) | 36.0 (15.5–51.2) |

**P*<0.05 versus baseline data; ^#^*P*<0.05 versus healthy individuals.
