# Supplementary figures and images for: p-STAT6, PU.1, and NF-κB are involved in allergen-induced late-phase airway inflammation in asthma patients
Source: BMC Pulm Med. 2015 Oct 14;15:122. doi: 10.1186/s12890-015-0119-7 (PMC4606997; doi:10.1186/s12890-015-0119-7)

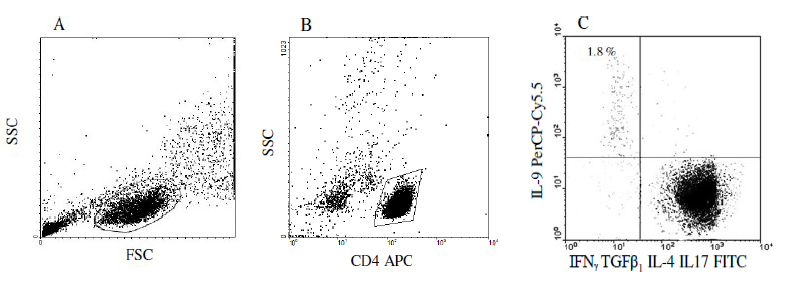

Supplement: Additional file 2: Figure S1. — Dot plot representing lymphocytes by size and granularity (A); T lymphocytes gated by expressing CD4+ (B); Th9 expression (C). (DOC 113 kb) [file 12890_2015_119_MOESM2_ESM.doc]

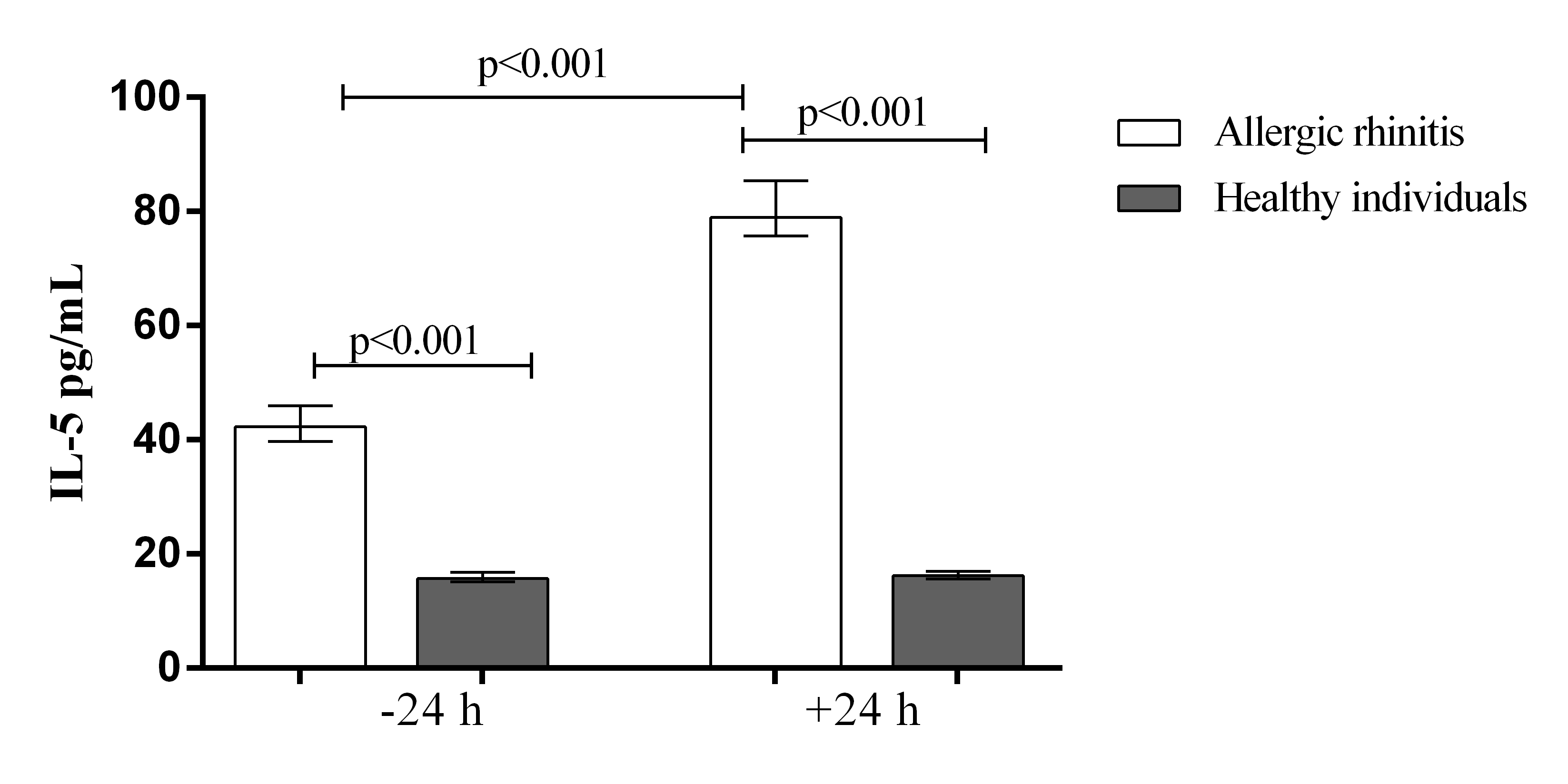


Healthy individuals

Supplement: Additional file 3: Figure S2. — The serum IL-5 concentration in patients with allergic asthma and healthy individuals before and after bronchial allergen challenge. (DOC 76 kb) [file 12890_2015_119_MOESM3_ESM.doc]
